# The Beneficial Effects of *Lacticaseibacillus paracasei* subsp. *paracasei* DSM 27449 in a Letrozole-Induced Polycystic Ovary Syndrome Rat Model

**DOI:** 10.3390/ijms25168706

**Published:** 2024-08-09

**Authors:** Yan Zhang Lee, Shih-Hsuan Cheng, Yu-Fen Lin, Chien-Chen Wu, Ying-Chieh Tsai

**Affiliations:** 1 Biomedical Industry Ph.D. Program, National Yang Ming Chiao Tung University, Taipei 112304, Taiwan; leannelee.ls09@nycu.edu.tw; 2 Bened Biomedical Co., Ltd., Taipei 115011, Taiwan; aibon03@hotmail.com (S.-H.C.); linyufen@benedbiomed.com (Y.-F.L.); allen@benedbiomed.com (C.-C.W.); 3 Institute of Biochemistry and Molecular Biology, National Yang Ming Chiao Tung University, Taipei 112304, Taiwan

**Keywords:** polycystic ovary syndrome, microRNAs, *Lacticaseibacillus paracasei* subsp. *paracasei*, letrozole, Bax

## Abstract

Polycystic ovary syndrome (PCOS) is a prevalent endocrine disorder affecting women of reproductive age globally. Emerging evidence suggests that the dysregulation of microRNAs (miRNAs) and gut dysbiosis are linked to the development of PCOS. In this study, the effects of *Lacticaseibacillus paracasei* subsp. *paracasei* DSM 27449 (DSM 27449) were investigated in a rat model of PCOS induced by letrozole. The administration of DSM 27449 resulted in improved ovarian function, reduced cystic follicles, and lower serum testosterone levels. Alterations in miRNA expressions and increased levels of the pro-apoptotic protein Bax in ovarian tissues were observed in PCOS-like rats. Notably, the administration of DSM 27449 restored the expression of miRNAs, including miR-30a-5p, miR-93-5p, and miR-223-3p, leading to enhanced ovarian function through the downregulation of Bax expressions in ovarian tissues. Additionally, 16S rRNA sequencing showed changes in the gut microbiome composition after letrozole induction. The strong correlation between specific bacterial genera and PCOS-related parameters suggested that the modulation of the gut microbiome by DSM 27449 was associated with the improvement of PCOS symptoms. These findings demonstrate the beneficial effects of DSM 27449 in ameliorating PCOS symptoms in letrozole-induced PCOS-like rats, suggesting that DSM 27449 may serve as a beneficial dietary supplement with the therapeutic potential for alleviating PCOS.

## 1. Introduction

Polycystic ovary syndrome (PCOS) is a reproductive and endocrine disorder that affects a significant portion of women of reproductive age, with a prevalence ranging between 5% and 20% [1]. The three classical features of PCOS are persistent menstrual irregularities, hyperandrogenism, and polycystic ovaries visible on ultrasound [2,3]. Women diagnosed with PCOS often exhibit metabolic disorders and reproductive abnormalities, as well as an increased risk of developing type 2 diabetes, dyslipidemia, cardiovascular disease, anxiety, and depression [4,5]. The etiology and pathophysiology of PCOS remain unknown; however, substantial evidence suggests that genetics, environmental factors, lifestyle factors, and dysbiosis of the gut microbiome can be responsible for the development of PCOS [6,7].

Most studies on PCOS have focused on metabolic parameters and sex hormone levels. However, numerous studies have demonstrated that the aberrant expression of microRNAs (miRNAs) is linked to human diseases, including diabetes, cardiovascular disease, neurodegenerative diseases, and various types of cancer [8,9]. Among these, aberrant miRNA–mRNA regulatory networks have been found in PCOS and may participate in the pathogenesis of PCOS [10]. The altered expression of miRNAs has often been detected in the plasma, serum, follicular fluid, and granulosa cells of patients with PCOS and animal models of PCOS [11,12]. Despite this, the impact of the dysregulation of miRNAs on PCOS pathogenesis remains to be elucidated.

The gut microbiome is a complex community composed of diverse bacteria, archaea, fungi, protozoa, viruses, and their metabolites, which all play a crucial role in host physiology [13]. In 2012, the theory of dysbiosis of the gut microbiota was initially proposed to emphasize the crucial role of the gut microbiome in the development of PCOS [7]. Since then, numerous studies have demonstrated that the gut microbiome might be associated with the occurrence and development of PCOS symptoms [13,14,15]. An increase in *Bacteroides*, *Escherichia/Shigella*, and *Streptococcus* and a decrease in *Akkermansia* and *Ruminococcaceae* were observed among patients with PCOS [14]. Guo et al. showed that in PCOS-like rats, testosterone and androstenedione levels were increased, accompanied by decreased levels of *Lactobacillus*, *Ruminococcus*, and *Clostridium* and increased levels of *Prevotella* [16]. Notably, by restoring the composition of the gut microbiome with *Lactobacillus* treatment or fecal microbiome transplantation from healthy rats, the symptoms of PCOS were alleviated [16]. Evidence suggests that probiotic interventions might be a feasible approach to relieve the symptoms of PCOS [17,18,19].

Probiotics are live microorganisms that, when administered in adequate amounts, confer health benefits on a host [20]. In general, the effects of probiotics are associated with the normalization of a perturbed microbiome and the production of useful metabolites or enzymes; however, some strain-specific effects at the intestinal or extraintestinal level can be found in certain probiotics [20]. For example, a study conducted by Zhang et al. demonstrated that the probiotic *Bifidobacterium lactis* V9 regulates the levels of sex hormones through the gut–brain axis in women with PCOS [19]. In another study, probiotic supplementation with *Lactobacillus acidophilus*, *Lacticaseibacillus casei*, and *Bifidobacterium bifidum* for 12 weeks had beneficial effects on both total testosterone level and total antioxidant capacity in women with PCOS [21]. Additionally, a combination of *Bifidobacterium*, *L. acidophilus*, and *Enterococcus faecalis* treatment restored the diversity of the gut microbiome and improved the reproductive and metabolic functions in 5α-dihydrotestosterone (DHT)-induced PCOS-like rats [18]. Similar to DHT, letrozole, a non-steroidal aromatase inhibitor, has been used to induce PCOS symptoms in rats, and this model is widely used for studying PCOS [22]. By blocking the conversion of androgen to estrogen, letrozole induces high androgen levels and symptoms similar to that of women with PCOS, such as acyclic menstruation, polycystic ovaries, an increased body weight and testosterone level, and insulin resistance [23].

To date, no medication has been specifically approved for the treatment of PCOS [24]; thus, the development of novel strategies to relieve PCOS symptoms is encouraged. In the present study, we investigated the beneficial effects of a specific strain, *Lacticaseibacillus paracasei* subsp. *paracasei* DSM 27449 (DSM 27449), on PCOS symptoms in a letrozole-induced PCOS-like rat model. We further analyzed hormones and biochemical profiles, miRNA profiles, and the gut microbiome to clarify the role of DSM 27449 in PCOS. We also investigated the correlations between the gut microbiome and PCOS-related parameters, as well as miRNA expressions. Therefore, the use of DSM 27449 as a health food or dietary supplement for PCOS should be further verified.

## 2. Results

### 2.1. DSM 27449 Alleviated Ovarian Dysfunction in Letrozole-Induced PCOS-like Rats

**Figure 1:**
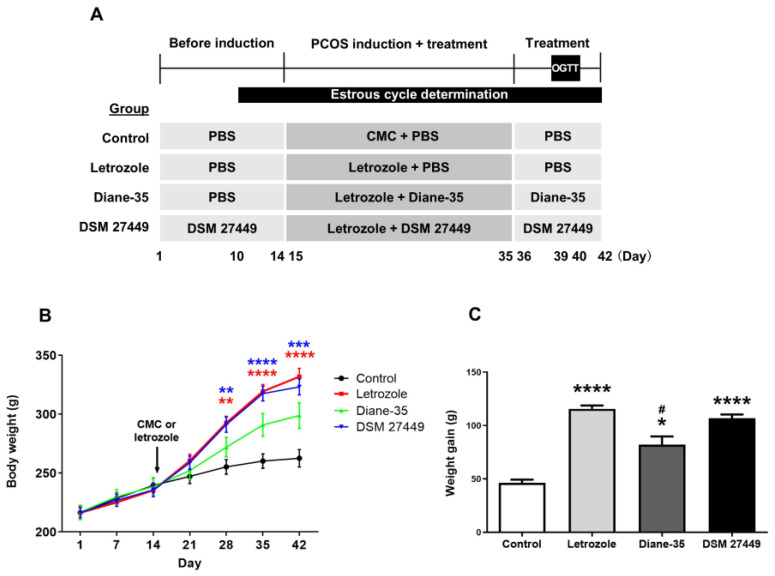
Effects of letrozole treatment on the weight of rats. (**A**) A detailed animal treatment scheme. Female Sprague-Dawley rats were randomly divided into four groups. The control group received 1% carboxymethyl cellulose (CMC), whereas the letrozole, Diane-35, and DSM 27449 groups received 1 mg/kg body weight of letrozole during polycystic ovary syndrome (PCOS) induction. Letrozole-induced PCOS-like rats received phosphate-buffered saline (PBS), Diane-35, or DSM 27449 by oral gavage. Estrous cycle determination was performed during the experiment, and an oral glucose tolerance test (OGTT) was performed at the end of the experiment. (**B**) Growth curves of rats from day 1–42. (**C**) Average weight gain at the end of the study between the groups. N = 8 per group; * *p* < 0.05, ** *p* < 0.01, *** *p* < 0.001, and **** *p* < 0.0001 compared with the control group; ^#^ *p* < 0.05 compared with the letrozole group.

To investigate the effects of DSM 27449 on PCOS symptoms, we utilized a letrozole-induced PCOS-like rat model. Letrozole, a non-steroidal aromatase inhibitor, was used to induce PCOS-like characteristics, because it reliably mimics the reproductive and metabolic features of PCOS in humans, including increased androgen levels, polycystic ovaries, irregular estrous cycles, and metabolic irregularities [23,25]. In this study, the rats were divided into four groups: control, letrozole, Diane-35, and DSM 27449. Except for the control group, all rats were induced with PCOS-like symptoms using letrozole. Diane-35, a combination of cyproterone acetate and ethinylestradiol, was applied as a treatment control, because it is commonly used to manage PCOS symptoms in clinical settings. It helps reduce androgen levels and improve menstrual regularity [22]. Diane-35 and DSM 27449 were administered to respective groups to evaluate their effects on PCOS-like symptoms. A schematic representation of the experimental design is shown in Figure 1A.

In all experimental groups, body weights were similar at the beginning of the experiment. Three weeks of letrozole treatment significantly increased the body weight of rats in the letrozole and DSM 27449 groups compared to that of those in the control group, while rats in the Diane-35 group showed a smaller increase in body weight (Figure 1B). Overall, the PCOS-like rats were significantly heavier than the control rats at the end of the study (Figure 1C, Control vs. Letrozole, *p* < 0.0001; Control vs. Diane-35, *p* = 0.0115; Control vs. DSM 27449, *p* < 0.0001). The administration of DSM 27449 did not significantly reduce the weight gain caused by PCOS induction. To comprehensively assess the metabolic and reproductive health of the rats, glucose homeostasis and insulin sensitivity were assessed, as PCOS is highly correlated with metabolic diseases. The PCOS-like rats exhibited higher fasting glucose levels than those in the control group (Figure S1A, Control vs. Letrozole, *p* = 0.0338; Control vs. Diane-35, *p* = 0.0035); however, the level of fasting glucose was comparable between the DSM 27449 and control groups (*p* = 0.4977). The levels of fasting insulin and the homeostasis model assessment of the insulin resistance index were significantly increased in the PCOS-like rats compared to the control group (Figure S1B,C, *p* < 0.0001), indicating that insulin resistance was observed in this PCOS animal model. Nevertheless, the administration of DSM 27449 and Diane-35 did not rescue insulin resistance. The oral glucose tolerance test (OGTT) results show no delay in glucose clearance between the groups during the test (Figure S1D,E). Additionally, the lipid profile, including the levels of triglycerides, total cholesterol, high-density lipoprotein (HDL) cholesterol, and low-density lipoprotein (LDL) cholesterol were examined, as lipid abnormalities are common in PCOS. The levels of these lipids were similar in the control, letrozole, and DSM 27449 groups (Figure S1F–I), indicating that letrozole treatment and DSM 27449 administration have no significant effects on the serum lipid profile in PCOS-like rats. Remarkably, HDL-cholesterol levels were the lowest in the Diane-35 group (Control vs. Diane-35, *p* = 0.0304).

**Figure 2:**
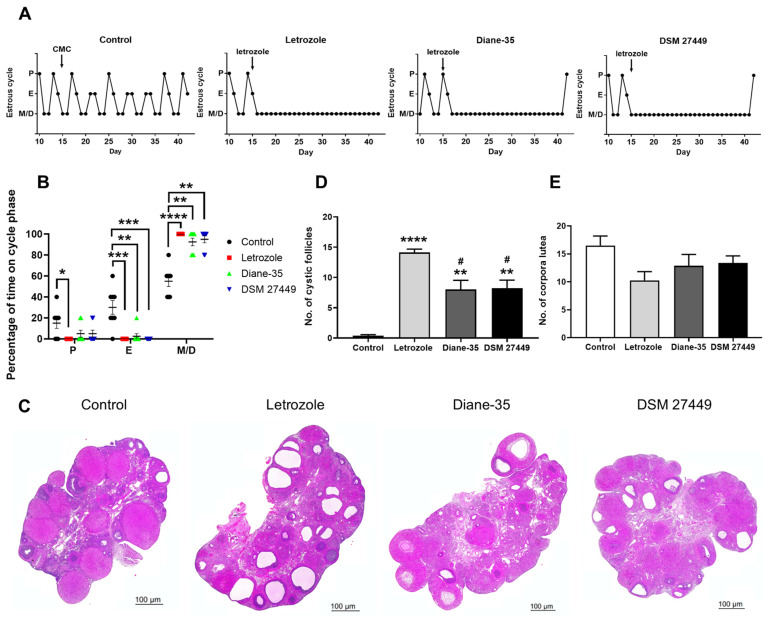
Effects of *L. paracasei* subsp. *paracasei* DSM 27449 on ovarian function in letrozole-induced polycystic ovary syndrome-like rats. (**A**) Representative estrous cycles in rats (P, proestrus; E, estrus; M/D, metestrus/diestrus). (**B**) Percentage of time spent in different phases of the estrous cycle for the last five days of the experiment. (**C**) Hematoxylin and eosin staining of representative ovaries. The number of (**D**) cystic follicles and (**E**) corpora lutea of the experimental groups. N = 8 per group; * *p* < 0.05, ** *p* < 0.01, *** *p* < 0.001, and **** *p* < 0.0001 compared with the control group; ^#^ *p* < 0.05 compared with the letrozole group. Scale bar = 100 μm in (**C**).

The reproductive health and ovarian function of the rats were evaluated by monitoring the estrous cycle and examining the ovarian morphology. The control rats exhibited regular estrous cycles of 4–5 days, whereas a majority of the PCOS-like rats were completely acyclic after the letrozole treatment (Figure 2A). Our results show that the PCOS-like rats spent a significantly higher percentage of time in the metestrus/diestrus phase than those in the control group, suggesting an irregular estrous cycle and reproductive dysfunction (Figure 2B, Control vs. Letrozole, *p* < 0.0001; Control vs. Diane-35, *p* = 0.0068; Control vs. DSM 27449, *p* = 0.0017). However, with the supplementation of DSM 27449, the disrupted estrous cycles improved, with 25% of the PCOS-like rats in the DSM 27449 group showing restored estrous cycles by the end of the experiment. A histological examination of the ovarian tissues showed multiple growing follicles at different developmental stages and a high number of corpora lutea in the control rats, whereas the PCOS-like rats exhibited an increased number of cystic follicles and fewer corpora lutea (Figure 2C). DSM 27449 significantly decreased the number of cystic follicles (Figure 2D, Letrozole vs. DSM 27449, *p* = 0.0135). A decreased number of corpora lutea was observed in the letrozole group, whereas DSM 27449 slightly recovered the number of corpora lutea compared to that in the letrozole group (Figure 2E). Overall, our data indicate that DSM 27449 has the potential to improve ovarian function and pathological changes, similar to the effects observed with Diane-35.

### 2.2. DSM 27449 Reduced the Levels of Serum Testosterone and Expressions of AR in Ovarian Tissues

**Figure 3:**
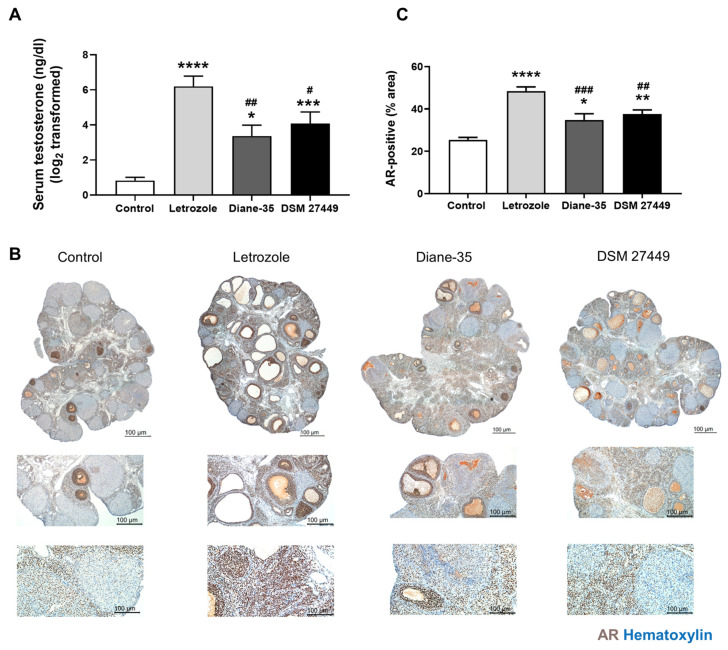
*L. paracasei* subsp. *paracasei* DSM 27449 attenuated an increase in serum testosterone levels and androgen-receptor (AR) expression in letrozole-induced polycystic ovary syndrome-like rats. (**A**) Serum testosterone levels. N = 6–8 per group. (**B**) AR immunohistochemical staining of representative ovarian sections. (**C**) Quantitative analysis of AR-positive areas in the ovarian sections. N = 8 per group; * *p* < 0.05, ** *p* < 0.01, *** *p* < 0.001, and **** *p* < 0.0001 compared with the control group; ^#^ *p* < 0.05, ^##^ *p* < 0.01, and ^###^ *p* < 0.001 compared with the letrozole group. Scale bar = 100 μm in (**B**).

An elevated level of testosterone is one of the typical characteristics of PCOS. In this study, serum testosterone levels were significantly higher in the letrozole group than those in the control group (Figure 3A, *p* < 0.0001). The administration of DSM 27449 and Diane-35 significantly reduced the testosterone levels compared to those in the letrozole group, returning them to levels similar to the control rats (Letrozole vs. DSM 27449, *p* = 0.0404; Letrozole vs. Diane-35, *p* = 0.0064). The androgen receptor (AR) is responsible for mediating the effects of androgens, and AR-mediated effects play a critical role in the development of PCOS [26]. Therefore, we further examined the expression of AR in ovarian tissues. An immunohistochemistry (IHC) analysis of AR expression demonstrated its presence in the ovarian tissue (Figure 3B). The average AR-positive areas in the control, letrozole, Diane-35, and DSM 27449 groups were 25.4%, 48.5%, 34.8%, and 37.6%, respectively. An increased immunoreactive signal was observed in the PCOS-like rats, but the AR-positive areas in the DSM 27449 and Diane-35 groups were significantly lower than those in the letrozole group (Figure 3C, Letrozole vs. DSM 27449, *p* = 0.0050; Letrozole vs. Diane-35, *p* = 0.0004).

### 2.3. DSM 27449 Restored the Dysregulated miRNA Expression in PCOS-like Rats

MiRNA dysregulation is often observed in patients with PCOS and PCOS animal models. To further clarify the role of miRNAs in PCOS, we evaluated the expression of a set of miRNAs that have been reported to be differentially expressed in PCOS, including miR-30a-5p, miR-93-5p, miR-223-3p, miR-320-3p, miR-324-3p, and miR-146a-5p, in the plasma and ovarian tissues of the rats [12,27,28,29,30]. We found that the expression of the plasma miR-30a-5p (*p* = 0.0335), miR-93-5p (*p* = 0.0469), and miR-223-3p (*p* = 0.0474) was significantly increased in the letrozole group compared to that in the control group (Table 1). The administration of DSM 27449 significantly reduced the expression of the plasma miR-30a-5p compared to that in the letrozole group (Letrozole vs. DSM 27449, *p* = 0.0066), and a decreasing trend in the expression of the plasmas miR-93-5p and miR-223-3p was observed. Similarly, the expression of miR-30a-5p was significantly increased in the ovarian tissues of the letrozole group (Control vs. Letrozole, *p* = 0.0227), whereas it was significantly reduced by DSM 27449 (Letrozole vs. DSM 27449, *p* = 0.0227). The expression levels of miR-320-3p, miR-324-3p, and miR-146a-5p in the plasma and ovarian tissues were comparable among all the groups. Our data demonstrate that DSM 27449 has the potential to restore the expression of miRNAs, as demonstrated by a significant increase in miR-30a-5p expression following letrozole induction, which was subsequently reduced by DSM 27449 administration in both the plasma and ovarian tissues.

### 2.4. Effects of DSM 27449 on the Expression of Bax, Bcl-2, and Beclin-1 in Ovarian Tissues

**Figure 4:**
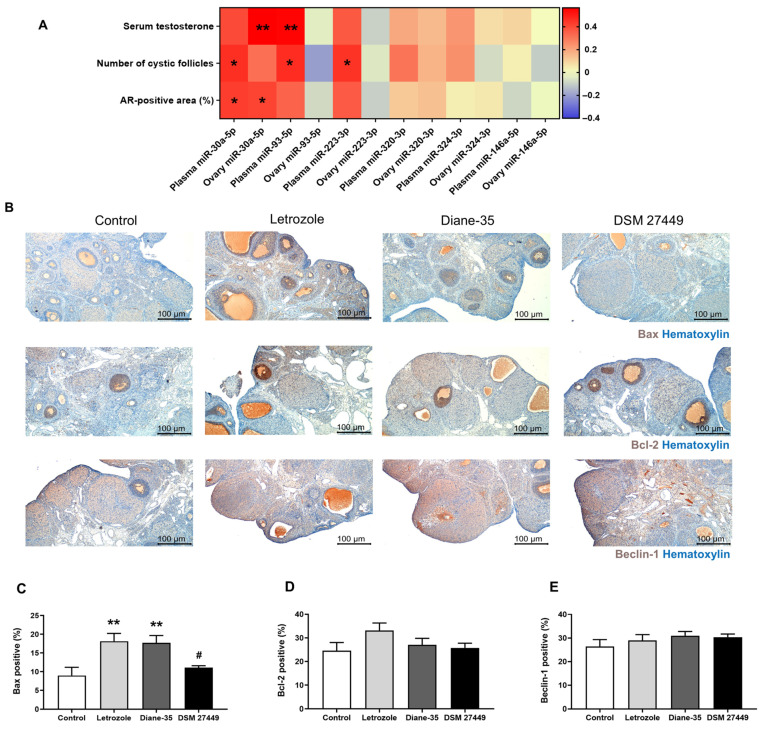
*L. paracasei* subsp. *paracasei* DSM 27449 downregulated the expression of the pro-apoptotic protein Bax in the ovarian tissues of letrozole-induced polycystic ovary syndrome (PCOS)-like rats. (**A**) Heat map of the Spearman’s rank correlation test visualizing the correlation between PCOS-related parameters and microRNA expressions. N = 6–8 per group, * *p* < 0.05 and ** *p* < 0.01. (**B**) Immunohistochemical staining of Bax (top), Bcl-2 (middle), and Beclin-1 (bottom) on representative ovarian sections. Quantitative analysis of the expression of (**C**) Bax, (**D**) Bcl-2, and (**E**) Beclin-1 in the ovarian sections. N = 8 per group; ** *p* < 0.01 compared with the control group; ^#^ *p* < 0.05 compared with the letrozole group. Scale bar = 100 μm in (**B**).

Next, we investigated the correlation between miRNA expressions and PCOS-related parameters, including the serum testosterone, number of cystic follicles, and AR-positive area in the ovarian tissue. We observed that miR-30a-5p, miR-93-5p, and miR-223-3p were significantly associated with PCOS-related parameters, suggesting that these miRNAs play critical roles in PCOS pathology (Figure 4A). Coincidentally, a dysregulation of these miRNAs and excess androgen production have been found to be involved in the stimulation and promotion of apoptosis and autophagy in ovarian cells [31,32,33]. Thus, we examined apoptosis and autophagy in the ovarian tissues by performing IHC staining for the pro-apoptotic protein Bax, anti-apoptotic protein Bcl-2, and autophagy-related protein Beclin-1. As shown in Figure 4B,C, the expression of Bax was significantly increased in the letrozole group compared to that in the control group (*p* = 0.0083), which shows a 2.02-fold increase. The administration of DSM 27449 significantly reduced the expression of Bax in the ovarian tissues compared to that in the letrozole group (Figure 4B,C, Letrozole vs. DSM 27449, *p* = 0.0443). Interestingly, Diane-35 treatment did not reduce Bax expressions, suggesting a different mechanism for ameliorating symptoms in PCOS-like rats. The expression levels of Bcl-2 and Beclin-1 were not significantly different between the groups (Figure 4D,E).

### 2.5. Diversity of the Gut Microbiome in Letrozole-Induced PCOS-like Rats

**Figure 5:**
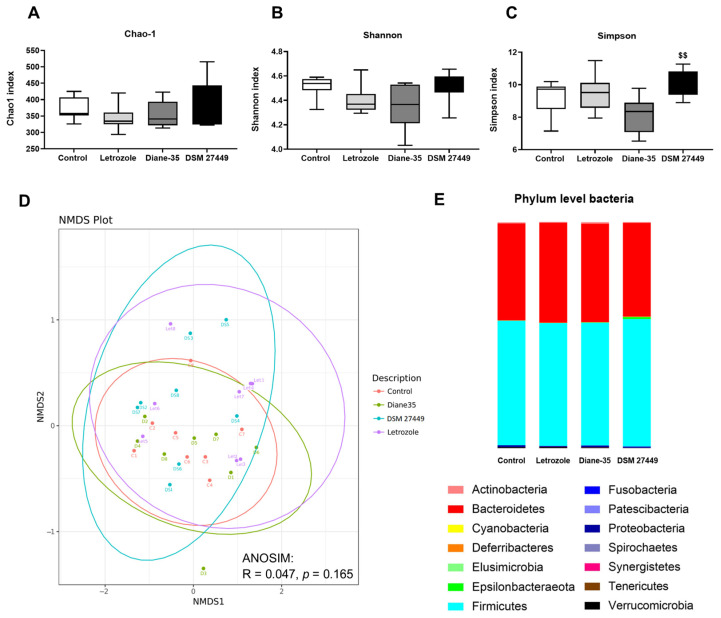

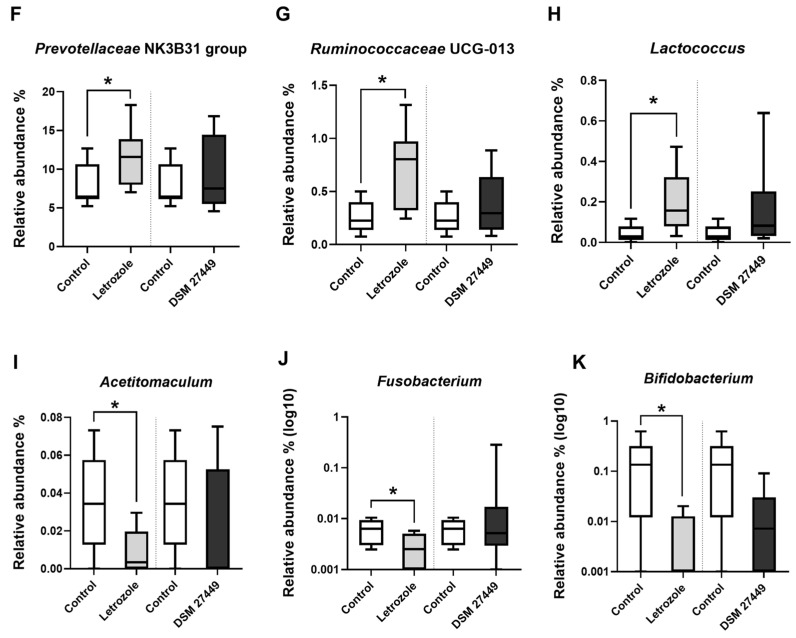
Effects of *L. paracasei* subsp. *paracasei* DSM 27449 on the gut microbiome in letrozole-induced polycystic ovary syndrome-like rats. Alpha diversity is represented by (**A**) the Chao1 index, (**B**) the Shannon index, and (**C**) the Simpson index. N = 8 per group; ^$$^ *p* < 0.01 compared with the Diane-35 group. (**D**) Beta diversity is represented by a non-metric multi-dimensional scaling (NMDS) plot and the analysis of similarities (ANOSIMs). (**E**) Composition of the gut microbiome at the phylum level. (**F**–**K**) Relative abundance of differential bacteria at the genus level between the control and letrozole groups and between the control and DSM 27449 groups. N = 8 per group; the Bonferroni method was applied to correct for Type I errors, with statistical significance considered at * *p* < 0.05/2.

Full-length 16S rRNA sequencing with a high quality and sufficient sequencing depth (Table S1) was performed to explore the effects of letrozole and DSM 27449 on the diversity and composition of the gut microbiome in PCOS-like rats. Alpha diversity was assessed using the Chao-1, Shannon, and Simpson indices to determine the species richness and evenness in the samples. No significant differences in alpha diversity were found among the control, letrozole, Diane-35, and DSM 27449 groups (Figure 5A–C). A non-metric multidimensional scaling (NMDS) plot based on weighted UniFrac distance matrices was generated to evaluate the differences in the beta diversity of the bacterial community. The results show no significant separation of the gut microbiome composition between the groups (Figure 5D, R = 0.047, *p* = 0.165). A linear discriminant analysis effect size was used to identify differentially expressed taxa in each group (Figure S2). Further analysis of the gut microbiome composition revealed 14 different taxonomic groups at the phylum level (Figure 5E). *Firmicutes* and *Bacteroidetes* were the dominant phyla, accounting for 97% of the reads. Although treatment with letrozole, Diane-35, or DSM 27449 had no significant effect on the alpha and beta diversity of the gut microbiome, we compared the relative abundance of the bacterial taxa at the genus level and identified six genera with differential expressions between the control and letrozole groups. Letrozole treatment significantly increased the relative abundances of the *Prevotellaceae* NK3B31 group (*p* = 0.0207), *Ruminococcaceae* UCG-013 (*p* = 0.0104), and *Lactococcus* (*p* = 0.0148) while decreasing the relative abundances of *Acetitomaculum* (*p* = 0.0193), *Fusobacterium* (*p* = 0.0204), and *Bifidobacterium* (*p* = 0.0056) compared to the control group (Figure 5F–K). Notably, the expression levels of these six genera were similar between the control and DSM 27449 groups, suggesting that DSM 27449 administration partially counteracted the alterations in the relative abundance induced by letrozole.

### 2.6. Correlation between Microbial Genera and PCOS-Related Parameters and Expression of miRNAs

**Figure 6:**
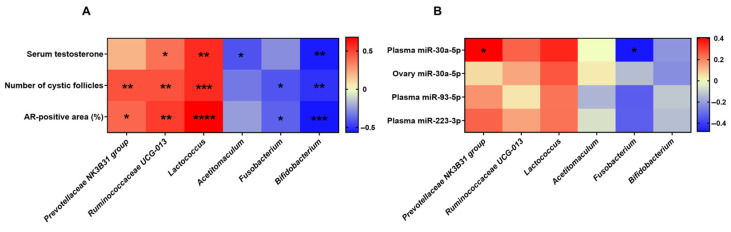
Heat maps of the Spearman’s rank correlation coefficient. (**A**) Heat map representing the correlation between differential genera and polycystic ovary syndrome-related parameters. (**B**) Heat map representing the correlation between differential genera and microRNA expressions. N = 6–8 per group; * *p* < 0.05, ** *p* < 0.01, *** *p* < 0.001, and **** *p* < 0.0001.

Spearman’s correlation analysis was used to investigate the correlation between differential microbial genera and the PCOS-related parameters. A majority of the microbial genera were significantly associated with the serum testosterone, number of cystic follicles, and AR-positive area in the ovarian tissues (Figure 6A). The *Prevotellaceae* NK3B31 group, *Ruminococcaceae* UCG-013, and *Lactococcus* were positively correlated with the PCOS-related parameters, whereas *Acetitomaculum*, *Fusobacterium*, and *Bifidobacterium* were negatively correlated with the PCOS-related parameters. These results indicate that the modulation of the microbial genera by DSM 27449 is positively associated with an improvement in PCOS symptoms.

We further assessed the association between microbial genera and plasma- and ovary-tissue-specific miRNAs across all cohorts (Figure 6B). The plasma miR-30a-5p demonstrated a statistically significant positive correlation with the *Prevotellaceae* NK3B31 group (r = 0.4065, *p* = 0.0318). Conversely, an inverse relationship was discerned between the plasma miR-30a-5p and *Fusobacterium* (r = −0.4777, *p* = 0.0101).

## 3. Discussion

PCOS is a common reproductive and endocrine disorder associated with multiple health issues in women of reproductive age. Insulin resistance, hyperinsulinemia, obesity, dyslipidemia, and metabolic disorders are often associated with PCOS [34,35]. In this study, the effects of DSM 27449 on the alleviation of the signs and symptoms in PCOS-like rats were investigated. An increased body weight and insulin resistance were observed in the letrozole-induced PCOS-like rats (Figure 1B,C and Figure S1C). These results are in line with those of a previous study, indicating that increased body weight might be partially caused by insulin resistance in PCOS-like rats [17]. The administration of DSM 27449 tends to result in lower fasting glucose levels in PCOS-like rats. However, DSM 27449 did not rescue insulin resistance or reduce the body weight. Additionally, DSM 27449 administration had no significant effect on the serum lipid profile of the PCOS-like rats. Therefore, we suggest that DSM 27449 exerts beneficial effects on PCOS-like rats in a way other than by regulating metabolic pathways.

A further investigation in reproductive health revealed that DSM 27449 administration improved irregular menstruation in the PCOS-like rats by restoring the estrous cycle, which had been prolonged in the diestrus phase by letrozole treatment. Similar to human PCOS, the ovaries of the PCOS-like rats showed an increased number of cystic follicles. DSM 27449 significantly reduced the number of cystic follicles and improved ovarian-morphology disruptions compared to those in the PCOS-like rats without treatment. The ovary is the main source of excess androgen production, and PCOS-related irregular menstruation is markedly influenced by androgen [33]. As the effects of testosterone are mediated through the interaction with its receptor—AR [36]—conceivably, the expression of AR was increased in the PCOS-like rats. This result is similar to that of a previous study that revealed that AR expression was higher in women with PCOS [37]. Our results demonstrate a significant reduction in the serum testosterone levels and expression of AR-positive areas following DSM 27449 administration. Thus, we suggest that DSM 27449 significantly improves ovarian dysfunction, probably by restoring abnormal testosterone levels and reducing the AR-positive areas in the ovarian tissues of PCOS-like rats (Figure 2 and Figure 3).

Next, we analyzed the expression of miRNAs, the dysregulation of which has been reported in previous studies on PCOS [11,30,38,39,40]. The miRNAs from the plasmas and ovaries were detected to better characterize their role in systemic circulation and in a tissue-specific manner. We observed that the administration of DSM 27449 significantly reduced the elevated expression of miR-30a-5p in both the plasma and ovarian tissues (Table 1). The expression of miR-30a-5p was found to be significantly upregulated in women with PCOS and in PCOS-like animal models in previous studies [30]. A previous study indicated that circulating miR-93 and miR-223 were higher in patients with PCOS, and women with PCOS can be differentiated from healthy controls by analyzing their miRNA levels [28]. Although the administration of DSM 27449 did not significantly reduce the expressions of miR-93-5p and miR-223-3p, it still showed a decreasing trend. Our results agree with those of the aforementioned studies that proposed an increased expression of miR-30a-5p, miR-93-5p, and miR-223-3p as biomarkers for PCOS diagnosis [28,41]. It is worth mentioning that the expressions of miR-30a-5p, miR-93-5p, and miR-223-3p were positively correlated with the serum testosterone level, number of cystic follicles, and AR-positive area in the ovarian tissue. Therefore, these miRNAs may be involved in the pathogenesis of PCOS. An abnormal expression of miR-30, miR-93, and miR-223 has been found to be related to aberrant steroidogenesis, insulin resistance, and the altered proliferation and/or apoptosis of ovarian cells in PCOS [11,41,42]. More recent work has demonstrated that miR-30a-5p could regulate the autophagy and apoptosis of granulosa cells while also being involved in producing steroid hormones and reactive oxygen species via targeting Beclin-1 [31]. Consistent with this, an elevated level of the autophagic marker, Beclin-1, was observed in the ovarian tissues of the PCOS-like rats [43]. Furthermore, miR-93 was involved in the maintenance of normal follicle development by targeting CDKN1A, which stimulates the cell growth and proliferation of granulosa cells, and/or targeting SMAD7, which mediates apoptosis in the ovary [32,44,45]. Similarly, miR-223-3p has been shown to be associated with the regulation of genes related to autophagy, such as FOXO1, FOXO3, Ataxin, and ATG7 [46]. Additionally, an excess of androgens can cause apoptosis, autophagy, mitochondrial dysfunction, and endoplasmic reticulum stress in the ovary, which in turn promotes PCOS [33]. Considering these, it is worthwhile to explore the effects of DSM 27449 supplementation on cell activities in the ovaries of PCOS-like rats.

The Bcl-2 protein family plays a vital role in the regulation of pro- and anti-apoptotic activities [47]. Alterations in the levels of proteins in this family, such as Bax (pro-apoptotic) and Bcl-2 (anti-apoptotic), have been shown to be associated with increased apoptosis in PCOS-like rats [48,49]. Based on our IHC staining results, the administration of DSM 27449 significantly reduced the upregulated expression of Bax induced by letrozole treatment (Figure 4). Thus, we suggest that DSM 27449 improves ovarian function by regulating apoptosis, possibly through the suppression of Bax. Notably, although DSM 27449 exerted positive outcomes similar to those of Diane-35 in the PCOS-like rats, we did not observe a reduction in Bax expression in the Diane-35 group. Therefore, Diane-35 and DSM 27449 may have distinct mechanisms of action despite producing similar beneficial effects. Autophagy is frequently observed in patients with PCOS and PCOS-like rats [50]. However, in our study, the expression of the essential autophagy protein, Beclin-1, was comparable in all the groups. Alterations in the levels of other autophagy-related proteins, such as LC3B and p62, should be further investigated to clarify the role of autophagy in PCOS-like rats. Overall, our results support the hypothesis that DSM 27449 reduces testosterone levels and AR expressions while downregulating specific miRNAs, such as miR-30a-5p, miR-93-5p, and miR-223-3p, which may participate in the regulation of ovarian apoptosis. Consequently, these effects ultimately alleviate PCOS-like symptoms.

Dysbiosis of the gut microbiome is a common finding in both human and animal models of PCOS [15]. A lower alpha diversity is often observed; however, in the case of beta diversity, Zhang et al. found a highly distinct difference in the intestinal microbiome between a control and PCOS group [19]. Conversely, Torres et al. revealed no distinct clustering of the microbiome composition between women with and without PCOS [51]. In our study, letrozole treatment did not have much impact on the alpha and beta diversity of the gut microbiome (Figure 5), and these results are in line with the findings of He and colleagues [17]. At the phylum level, *Firmicutes* and *Bacteroidetes* were abundant and dominated in the gut composition among different groups, confirming previous observations [52]. Despite not seeing significant differences in the microbial taxa, we observed changes in the relative abundance of certain genera between the groups (Figure 5). Letrozole treatment significantly induced alterations in the microbiome composition, including the *Prevotellaceae* NK3B31 group, *Ruminococcaceae* UCG-013, *Lactococcus*, *Acetitomaculum*, *Fusobacterium*, and *Bifidobacterium*, which were subsequently modulated by the intervention with DSM 27449. An increase in *Ruminococcaceae* was previously linked to an excess of androgens and metabolic disorders [53,54,55,56]. In our study, *Ruminococcaceae* UCG-013 positively correlated with the PCOS-related parameters, including the serum testosterone, number of cystic follicles, and AR-positive area in the ovarian tissue (Figure 6A). The genus *Lactococcus* has been found to be significantly enriched in rats with a prenatal exposure to an excess of androgens [55], suggesting a link between *Lactococcus* and androgen excess. Similar to a previous study, an increased abundance of *Lactococcus* was observed in PCOS-like rats, and *Lactococcus* was also found to be positively associated with the testosterone levels in our study. *Acetitomaculum* is a type of bacteria that produces short-chain fatty acids (SCFAs), and its higher relative abundance has been correlated with higher levels of acetate detected in the cecum contents of rats [57,58]. Interestingly, a significant reduction in the levels of SCFAs in patients with PCOS has been reported [19]. Therefore, the decreased abundance of *Acetitomaculum* in the PCOS-like rats may be partly responsible for the impact on SCFA production and the host metabolism. *Bifidobacterium* is a beneficial microbe involved in the maintenance of a balanced gut microbiome and can enhance immunity and nutrient absorption [59]. In the present study, the genus *Bifidobacterium* was detected with a lower abundance in the PCOS-like rats, and this observation is consistent with a previous study [19]. The genus *Fusobacterium* has been formerly detected with a significantly higher abundance in obese patients with PCOS [60]. *Fusobacterium* is a proinflammatory pathogen, and a higher abundance was found in patients with inflammatory bowel disease, colorectal adenomas, and other diseases [60,61,62]. Nonetheless, our results indicate that the relative abundance of *Fusobacterium* was significantly reduced in the PCOS-like rats. The relative abundance of the *Prevotellaceae* NK3B31 group significantly increased after the letrozole treatment; although the *Prevotellaceae* NK3B31 group has not been shown to be related to PCOS, it has been associated with cortisol levels and higher proinflammatory cytokines [63].

When correlating the differential taxa with PCOS-related parameters in all study groups, most genera were found to be highly correlated with the serum testosterone, number of cystic follicles, and AR-positive area in the ovarian tissue (Figure 6A). Our results indicate that these genera are associated with PCOS and its symptoms. Hence, the beneficial effects of DSM 27449 on PCOS symptoms may be partially attributed to the modulation of an altered gut microbiome. Furthermore, the *Prevotellaceae* NK3B31 group showed a positive correlation, whereas *Fusobacterium* showed a negative correlation with the expression of the plasma miR-30a-5p (Figure 6B). This finding is intriguing, because, to the best of our knowledge, neither *Prevotellaceae* NK3B31 nor *Fusobacterium* has been shown to be associated with miRNA expressions. Current studies demonstrate the ability of probiotics to influence the gut microbiome and regulate specific genes via the modulation of miRNAs [64]. According to a study conducted by Vahed et al., *Leuconostoc mesenteroides* has demonstrated the ability to induce apoptosis in colon cancer cell lines by modulating the expression of the oncomiRNAs miR-200b and miR-21, pro-apoptotic factors, and cell survival proteins [65]. It is possible that DSM 27449 supplementation alleviates PCOS symptoms through a similar mechanism. Despite this, our understanding of the relationship between the gut microbiome and miRNA expressions remains underexplored and should be further studied in the future.

One limitation of our study is the absence of an additional control group of rats supplemented with DSM 27449 without the induction of PCOS symptoms. While we did not explore the impact of DSM 27449 on healthy rats, it is important to note that its administration in PCOS-induced rats did not result in any adverse effects or deaths. Subsequent studies should address this gap to gain a more comprehensive understanding of DSM 27449’s potential benefits and safety profile in various physiological contexts. Additionally, our study primarily focused on the metabolic and endocrine parameters of PCOS, but we did not investigate the effects of DSM 27449 on fertility in the rat models. Future research should explore this aspect to fully understand the potential of DSM 27449 in addressing all symptoms of PCOS, including infertility. Our current analysis of the bacterial composition using U tests with Bonferroni corrections also has limitations. Advanced tools like MaAsLin2 or LinDA should be employed to enhance the accuracy and reliability of microbiome analyses, providing more robust and in-depth information [66,67].

## 4. Materials and Methods

### 4.1. Preparation of DSM 27449

DSM 27449 was isolated from a healthy individual. Sub-culturing of DSM 27449 was performed according to previous studies [68,69]. Briefly, DSM 27449 was cultured in a de Man Rogosa Sharpe (MRS) broth (Becton-Dickinson, Sparks, MD, USA) at 37 °C for 18 h. After cultivation, the culture was harvested by centrifugation at 8000× *g* for 15 min. The supernatant was discarded, and the resulting pellet was resuspended in an MRS broth containing 20% glycerol. The suspension was then aliquoted into tubes and stored at −20 °C until use. For daily preparation, frozen aliquots of DSM 27449 were thawed and washed with a phosphate-buffered saline (PBS). The concentration was adjusted to 1 × 10^9^ colony-forming units (CFUs)/mL in PBS before oral administration.

### 4.2. Animals and Treatments

Eight-week-old female Sprague-Dawley rats (BioLASCO Taiwan Co., Ltd., Taipei City, Taiwan) were purchased and housed under standard laboratory conditions with ad libitum access to food and water. This study complied with the ARRIVE guidelines, and all animal procedures were approved by the Institutional Animal Care and Use Committee of the National Yang Ming Chiao Tung University (protocol number: 1100802). The rats were divided into four groups (n = 8 per group): the control, letrozole, Diane-35, and DSM 27449 groups. The non-steroidal aromatase inhibitor letrozole (Cayman Chemical, Ann Arbor, MI, USA) was used to induce PCOS-like symptoms [25]. The PCOS-like rats received an oral administration of 1 mg/kg body weight of letrozole dissolved in 1% carboxymethyl cellulose (CMC) (Fujifilm Wako Pure Chemical Corporation, Osaka, Japan), while the control rats received 0.5 mL of 1% CMC for three consecutive weeks [25]. Diane-35 (Bayer Schering Pharma, Leverkusen, Germany) was prepared as one tablet suspended in 25 mL of 1% CMC and administered at a dosage of 4.5 mL/kg to rats in the treatment control group. DSM 27449 (1 × 10^9^ CFU/mL) was administered to the rats prior to PCOS induction and throughout the study period. The estrous cycle was monitored, and OGTT was performed. At the end of the experiment, all the rats were sacrificed for subsequent analyses.

### 4.3. Determination of Estrous Cycle Phases

Phases of the estrous cycle were determined by predominant cell types in vaginal smears [70]. Briefly, approximately 150–200 μL of a PBS was flushed into the rats’ vagina and back out two or three times to collect vaginal cells for microscopic examination [71]. The estrous cycle was classified into four phases (proestrus, estrus, metestrus, and diestrus), and the phases were identified based on the relative proportion of nucleated epithelial cells, anucleated keratinized epithelial cells, and leukocytes [72].

### 4.4. Ovarian Morphology

After the rats were sacrificed, their ovaries were harvested and immediately fixed in 10% formalin (JT Baker, Phillipsburg, NJ, USA). The ovaries were sent to the National Laboratory Animal Center (Taipei City, Taiwan) for the preparation of microscope slides with hematoxylin and eosin-stained histological sections for evaluation. The number of cystic follicles and corpora lutea were evaluated by two blinded observers.

### 4.5. Hormones and Biochemical Profile

To obtain the serum, blood samples were collected into serum-separator tubes (BD Vacutainer^®^, Becton, Dickinson and Company, Franklin Lakes, NJ, USA); placed at room temperature for 0.5 h; and centrifuged at 1500× *g* for 15 min. To obtain the plasmas, blood samples were collected into tubes that contained dipotassium ethylenediaminetetraacetic acid (EDTA) (BD Vacutainer^®^, Becton, Dickinson and Company) and centrifuged at 1500× *g* for 15 min. The serum or plasmas were collected, aliquoted, and stored at −80 °C for further analysis. Testosterone and HDL-cholesterol and LDL-cholesterol levels were measured using the Elecsys assay on Cobas immunoassay analyzers (Roche Diagnostics, Meylan, France). Glucose, triglyceride, and total cholesterol levels were measured using the Dri-Chem NX600 equipment (Fujifilm, Tokyo, Japan). Insulin levels were measured using an enzyme-linked immunosorbent assay kit (Cusabio, Houston, TX, USA).

### 4.6. IHC Analysis

IHC staining was conducted to evaluate the expression of AR, apoptosis-related proteins (Bax and Bcl-2), and autophagy-related protein (Beclin-1) in ovarian tissues. Ovarian sections were subjected to antigen retrieval in an EDTA solution for 90 min. The sections were then incubated with primary antibodies against AR (760-4605, Cell Marque Corporation, Rocklin, CA, USA); Bax (1:300 dilution, sc-7480, Santa Cruz Biotechnology Inc., Santa Cruz, CA, USA); Bcl-2 (790-4604, Ventana Medical Systems Inc., Tucson, AZ, USA); and Beclin-1 (1:50 dilution, sc-48341, Santa Cruz Biotechnology) for 2 h. The sections were washed, incubated with a specific secondary antibody for 1.5 h, and counterstained with hematoxylin. The slides were scanned, and positive staining was quantified using the ImageJ software (version 1.4.3.67, National Institutes of Health, Bethesda, MD, USA).

### 4.7. Profiling of miRNA Expressions

The ovarian tissues were harvested and stored at −80 °C until analysis. For RNA isolation from the ovaries, the total RNA was extracted from 20–30 μg of tissues using the RNeasy Mini Kit (Qiagen, Hilden, Germany), according to the manufacturer’s protocol. For RNA isolation from the plasmas, RNA fragments of <1000 nucleotides were isolated from 100 μL of the plasma samples using the LiqmiR microRNA Extraction Kit (Topgen Biotech, Kaohsiung City, Taiwan), according to the manufacturer’s instructions. The RNA isolated from the ovaries and plasmas was stored at −80 °C for further use. A complementary DNA synthesis was performed using the miRscript RT Kit (Topgen Biotech) based on reverse transcription with the specific miRNA stem-loop reverse-transcribed primers listed in Table S2. A real-time polymerase chain reaction (PCR) was performed on a StepOnePlus Real-Time PCR system (Applied Biosystems, Foster City, CA, USA) using the TopGE Probe qPCR Master Mix and TopQ miRNA probe qPCR assay for rno-miR-30a-5p, rno-miR-93-5p, rno-miR-223-3p, rno-miR-320-3p, rno-miR-324-3p, rno-miR-146a-5p, and U6 snRNA or cel-miR-39-3p (Topgen Biotech). Amplification data were normalized to U6 snRNA and cel-miR-39-3p expressions in the ovarian and plasma samples, respectively. The relative expression was quantified using the 2^-∆∆Ct^ relative quantification method [73].

### 4.8. Extraction of Bacterial DNA

Fresh fecal samples were collected into sterile tubes with the RNAlater Stabilization Solution (Thermo Fisher Scientific, Vienna, Austria) and stored at −80 °C for further analysis. Microbial DNA was extracted and purified using previously described techniques [69]. Briefly, a DNA-extraction buffer, glass beads, and phenols were added to the fecal mixture, homogenized, and centrifuged at 12,000× *g* for 5 min. The supernatants were collected for phenol-chloroform extraction and ethanol precipitation. The extracted DNA was dissolved in 60 μL of nuclease-free water and stored at −80 °C.

### 4.9. 16S rRNA Sequencing and Bioinformatic Analysis

The full-length 16S rRNA gene (V1-V9) was amplified using the bacterial universal primer sets 27F: AGRGTTYGATYMTGGCTCAG and 1492R: RGYTACCTTGTTACGACTT, with PacBio barcodes in the PCR system. The PCR products were examined using 0.8% agarose gels and the Qubit 2.0 Fluorometer (Thermo Scientific, Waltham, MA, USA). Qualified PCR products were purified using AMPure PB beads (PacBio, Menlo Park, CA, USA) after mixing in equal amounts. The 16S rRNA amplicons were pooled and used to create an SMRTbell library. Single-molecule real-time (SMRT) sequencing was performed using a PacBio Sequel IIe sequencer with Chemistry version 2 (Pacific Biosciences, Menlo Park, CA, USA). After primary filtering on the Sequel IIe System, a secondary analysis was conducted using the SMRT analysis pipeline version 11.0.0 (Pacific Biosciences). A PacBio Sequel sequencer was used to sequence amplicon libraries (Genomics BioSci & Tech Co., New Taipei City, Taiwan). Sub-reads were transferred to circular consensus sequencing reads and demultiplexed. The filtered sequences were clustered into operational taxonomic units at 99% identity using the Mothur (version 1.44.0) software with the SILVA database (SILVA 132) and further analyzed using QIIME (version 1.9.0). An alpha-diversity analysis was performed using a species-richness estimator (Chao1) and species-evenness estimators (Shannon and Simpson). Beta diversity was analyzed using NMDS. The analysis of similarities, a non-parametric multivariate analysis, was applied to calculate the ranked dissimilarity matrix. The Mann–Whitney U test was employed to distinguish variations in the relative abundance of microbes, and the Bonferroni method was applied to correct for Type I errors.

### 4.10. Statistical Analysis

The software GraphPad Prism 9 (GraphPad Software Inc., La Jolla, CA, USA) was used to process the experimental data, and all data are shown as a mean ± the standard error of the mean. Between-group differences were analyzed using a one-way or two-way analysis of variance followed by Tukey’s post-hoc test, Welch ANOVA followed by Dunnett’s T3 multiple comparisons test, or the Kruskal–Wallis test followed by Dunn’s multiple comparisons test, depending on the data distribution and assumption requirements. Spearman’s correlation coefficient was used for a correlation analysis of miRNA expressions and PCOS-related parameters, microbial genera and PCOS-related parameters, and microbial genera and miRNA expressions. The non-parametric Mann–Whitney U test was used to evaluate statistical significance in the relative abundance of bacteria between two groups, and the Bonferroni method was applied to correct for Type I errors.

## 5. Conclusions

The present study demonstrates the beneficial effects of a novel strain, *L. paracasei* subsp. *paracasei* DSM 27449, on the amelioration of PCOS-like symptoms induced by letrozole in a rat model. The administration of DSM 27449 significantly improves ovarian function and reduces serum testosterone levels and AR expressions in the ovarian tissue. By regulating the expressions of miR-30a-5p, miR-93-5p, and miR-223-3p, DSM 27449 downregulates the level of the pro-apoptotic protein Bax in ovarian tissues and eventually improves PCOS symptoms. The genera that were differentially expressed in PCOS-like rats, such as the *Prevotellaceae* NK3B31 group, *Ruminococcaceae* UCG-013, *Lactococcus*, *Acetitomaculum*, *Fusobacterium*, and *Bifidobacterium*, can serve as microbial signatures in PCOS disorders. The effects of DSM 27449 on the modulation of differential genera in PCOS-like rats may contribute to the alleviation of PCOS symptoms. Further studies are required to clarify the relationship between PCOS, miRNA expression, and the gut microbiome. Our study provides data supporting the use of probiotics, such as DSM 27449, as food supplements in the amelioration of PCOS.

## Figures and Tables

**Table 1:** Effects of *L. paracasei* subsp. *paracasei* DSM 27449 on the expression levels of microRNAs (miRNAs) in plasmas and ovaries.

miRNA	Source	Control	Letrozole	Diane-35	DSM 27449
miR-30a-5p	Plasma	1.029 ± 0.10	1.629 ± 0.21 *	0.771 ± 0.08 ^##^	0.886 ± 0.15 ^##^
	Ovary	1.075 ± 0.05	1.475 ± 0.13 *	1.188 ± 0.10	1.075 ± 0.06 ^#^
miR-93-5p	Plasma	1.363 ± 0.19	2.557 ± 0.48 *	1.557 ± 0.23	1.843 ± 0.28
	Ovary	1.125 ± 0.08	1.100 ± 0.06	0.925 ± 0.07	0.900 ± 0.08
miR-223-3p	Plasma	1.386 ± 0.29	2.671 ± 0.45 *	1.800 ± 0.22	1.838 ± 0.29
	Ovary	0.825 ± 0.07	0.888 ± 0.24	1.038 ± 0.21	0.825 ± 0.13
miR-320-3p	Plasma	3.538 ± 1.53	15.78 ± 8.71	3.043 ± 0.79	4.275 ± 1.00
	Ovary	1.513 ± 0.30	2.013 ± 0.41	2.738 ± 0.87	1.450 ± 0.17
miR-324-3p	Plasma	2.638 ± 0.62	3.775 ± 0.75	4.757 ± 0.90	4.200 ± 0.97
	Ovary	1.138 ± 0.08	1.263 ± 0.11	1.088 ± 0.07	1.013 ± 0.08
miR-146a-5p	Plasma	1.750 ± 0.28	2.413 ± 0.48	2.271 ± 0.52	2.500 ± 0.60
	Ovary	0.913 ± 0.09	1.063 ± 0.24	1.013 ± 0.14	0.775 ± 0.14

Relative expression of miRNAs in plasmas and ovaries. N = 7–8 per group; * *p* < 0.05 compared with the control group; ^#^ *p* < 0.05 and ^##^ *p* < 0.01 compared with the letrozole group.

## Data Availability

Data are included in the article/Supplementary Materials.

## Supplementary Materials

The following supporting information can be downloaded at: https://www.mdpi.com/article/10.3390/ijms25168706/s1.
