# Supplementary material for: The Beneficial Effects of Lacticaseibacillus paracasei subsp. paracasei DSM 27449 in a Letrozole-Induced Polycystic Ovary Syndrome Rat Model
Source: Int J Mol Sci. 2024 Aug 9;25(16):8706. doi: 10.3390/ijms25168706 (PMC11354393; doi:10.3390/ijms25168706)
Supplement: Supplementary file 1 [file ijms-25-08706-s001.zip › ijms-3102132-supplementary.pdf]

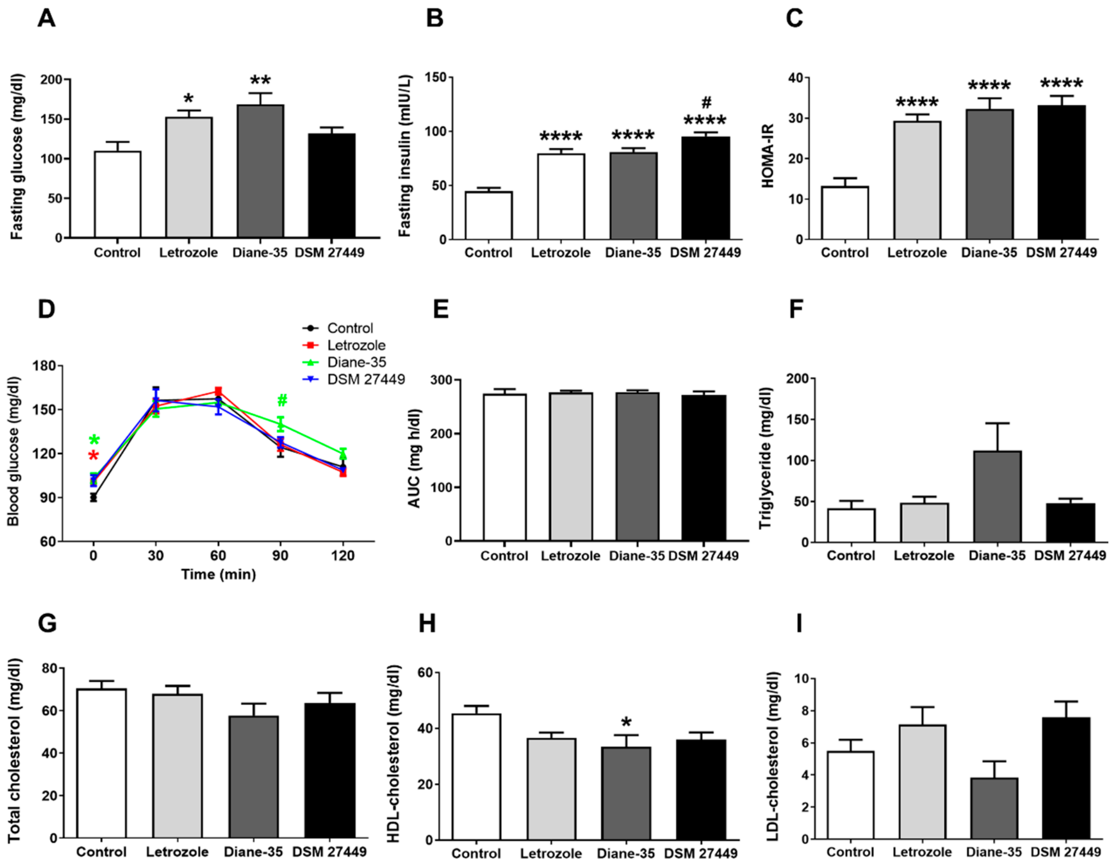

Figure S1. Effects of *L. paracasei* subsp. *paracasei* DSM 27449 on glucose tolerance and serum lipid profile in letrozole-induced PCOS-like rats. (**A**) Fasting blood glucose and (**B**) fasting insulin levels in the rats. (**C**) The homeostasis model assessment of insulin resistance (HOMA-IR) index was calculated using the formula: [fasting glucose levels (mmol/L)] * [fasting insulin (mlU/mL)]/22.5. (**D**) Blood glucose levels from oral glucose tolerance test (OGTT). (**E**) The area under the curve (AUC) of blood glucose levels was calculated. The levels of (**F**) triglyceride, (**G**) total cholesterol, (**H**) high-density lipoprotein (HDL)-cholesterol, and (**I**) low-density lipoprotein (LDL)-cholesterol. N = 6–7 per group, * $p < 0.05$, ** $p < 0.01$, **** $p < 0.0001$ compared with the control group; $^{\#}$ $p < 0.05$ compared with the letrozole group.

A

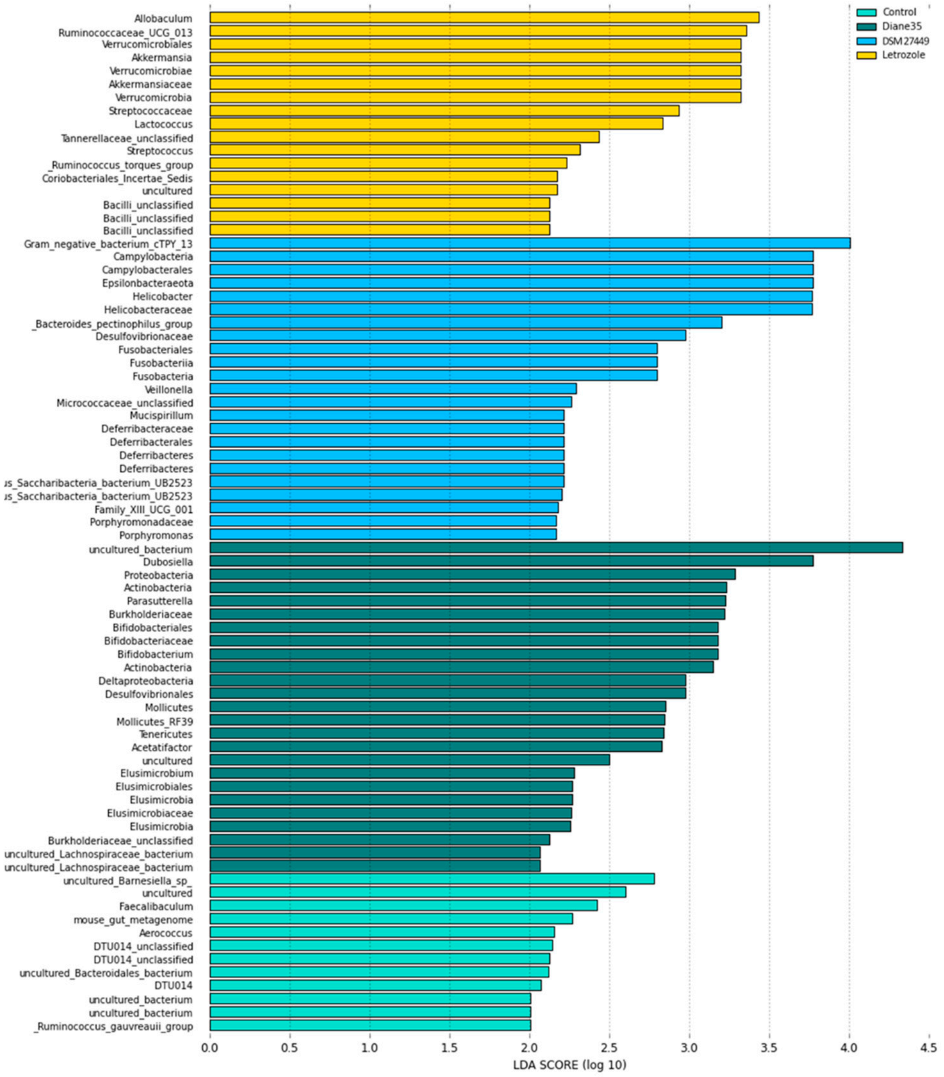

Control
Diane35
DSM 27449
Letrozole
Allobaculum
Ruminococcaceae_UCG_013
Verrucomicrobiales
Akkermansia
Verrucomicrobiae
Akkermansiaceae
Verrucomicrobia
Streptococcaceae
Lactococcus
Tannerellaceae_unclassified
Streptococcus
_Ruminococcus_torques_group
Coriobacteriales_Incertae_Sedis
uncultured
Bacilli_unclassified
Bacilli_unclassified
Bacilli_unclassified
Gram_negative_bacterium_cTPY_13
Campylobacteria
Campylobacterales
Epsilonbacteraeota
Helicobacter
Helicobacteraceae
_Bacteroides_pectinophilus_group
Desulfovibrionaceae
Fusobacteriales
Fusobacteriia
Fusobacteria
Veillonella
Micrococcaceae_unclassified
Mucispirillum
Deferribacteraceae
Deferribacterales
Deferribacteres
Deferribacteres
s_Saccharibacteria_bacterium_UB2523
s_Saccharibacteria_bacterium_UB2523
Family_XIII_UCG_001
Porphyromonadaceae
Porphyromonas
uncultured_bacterium
Dubosiella
Proteobacteria
Actinobacteria
Parasutterella
Burkholderiaceae
Bifidobacteriales
Bifidobacteriaceae
Bifidobacterium
Actinobacteria
Deltaproteobacteria
Desulfovibrionales
Mollicutes
Mollicutes_RF39
Tenericutes
Acetatifactor
uncultured
Elusimicrobium
Elusimicrobiales
Elusimicrobia
Elusimicrobiaceae
Elusimicrobia
Burkholderiaceae_unclassified
uncultured_Lachnospiraceae_bacterium
uncultured_Lachnospiraceae_bacterium
uncultured_Barnesiella_sp_
uncultured
Faecalibaculum
mouse_gut_metagenome
Aerococcus
DTU014_unclassified
DTU014_unclassified
uncultured_Bacteroidales_bacterium
DTU014
uncultured_bacterium
uncultured_bacterium
_Ruminococcus_gauvreauii_group
0.0 0.5 1.0 1.5 2.0 2.5 3.0 3.5 4.0 4.5
LDA SCORE (log 10)

B

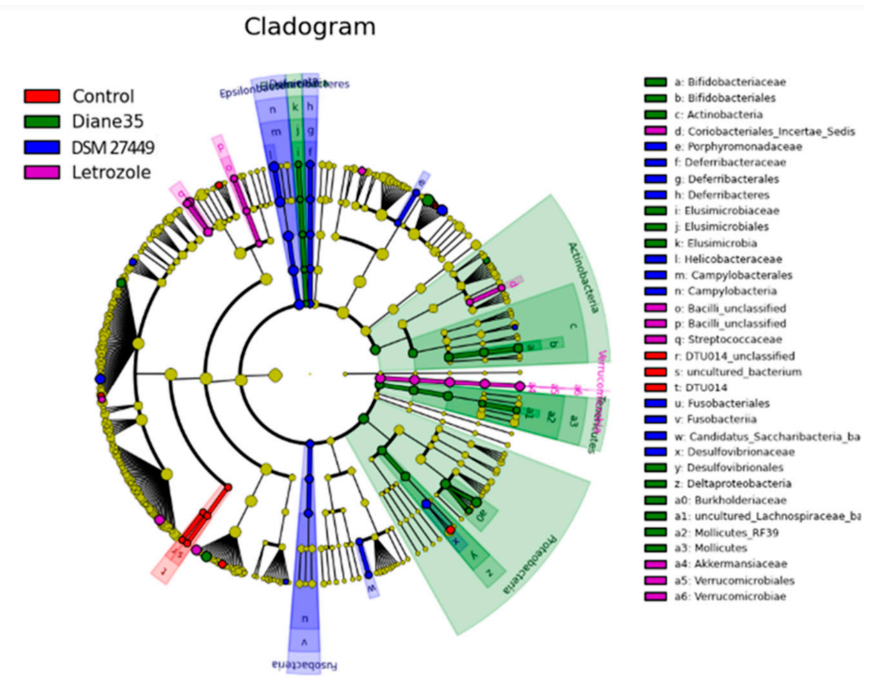

Cladogram
Control
Diane35
DSM 27449
Letrozole
Actinobacteria
Proteobacteria
Fusobacteria
a: Bifidobacteriaceae
b: Bifidobacteriales
c: Actinobacteria
d: Coriobacteriales_Incertae_Sedis
e: Porphyromonadaceae
f: Deferribacteraceae
g: Deferribacterales
h: Deferribacteres
i: Elusimicrobiaceae
j: Elusimicrobiales
k: Elusimicrobia
l: Helicobacteraceae
m: Campylobacterales
n: Campylobacteria
o: Bacilli_unclassified
p: Bacilli_unclassified
q: Streptococcaceae
r: DTU014_unclassified
s: uncultured_bacterium
t: DTU014
u: Fusobacteriales
v: Fusobacteriia
w: Candidatus_Saccharibacteria_ba
x: Desulfovibrionaceae
y: Desulfovibrionales
z: Deltaproteobacteria
a0: Burkholderiaceae
a1: uncultured_Lachnospiraceae_ba
a2: Mollicutes_RF39
a3: Mollicutes
a4: Akkermansiaceae
a5: Verrucomicrobiales
a6: Verrucomicrobiae

Figure S2. Differential microbiome composition of fecal specimens from control, letrozole, Diane-35, and DSM 27449 groups. (**A**) Bar plot and (**B**) cladogram of linear discriminant analysis effect size analysis showed the effect size for a particular taxon in a certain group. Only the taxa with linear discriminant analysis (LDA) scores >2 are displayed.

Table S1. Summary of 16S rRNA sequencing data quality

Sample name	% GC	Sequence length (bp)	Total sequences	Unique tags	No. of OTUs	Good's coverage
Control-1	55	1,484	34800	29608	23020	0.998
Control-2	55	1,483	44300	37270	28749	0.999
Control-3	55	1,489	29000	23044	17615	0.997
Control-4	54	1,480	31100	25758	19091	0.997
Control-5	55	1,489	33200	28300	22728	0.998
Control-6	55	1,490	30400	25374	19930	0.998
Control-7	55	1,481	38500	30798	22488	0.998
Control-8	55	1,486	41600	34672	27322	0.998
Letrozole-1	54	1,494	43200	32362	23119	0.998
Letrozole-2	54	1,490	50800	37186	25779	0.999
Letrozole-3	54	1,490	31800	23787	17280	0.998
Letrozole-4	55	1,493	36800	27687	19989	0.998
Letrozole-5	55	1,485	33700	28685	22708	0.998
Letrozole-6	55	1,487	35700	31029	24849	0.998
Letrozole-7	55	1,496	41100	30632	22151	0.998
Letrozole-8	55	1,480	40700	34262	26614	0.998
Diane-35-1	55	1,501	42000	31346	22709	0.998
Diane-35-2	56	1,484	48500	40511	30615	0.999
Diane-35-3	54	1,503	29200	21397	15788	0.998
Diane-35-4	55	1,486	30900	26945	21293	0.998
Diane-35-5	55	1,483	29100	24329	18737	0.998
Diane-35-6	55	1,477	26600	20837	14282	0.997
Diane-35-7	55	1,487	36700	29232	21869	0.997
Diane-35-8	55	1,486	43600	36349	28495	0.998
DSM 27449-1	55	1,488	37500	30132	23077	0.998
DSM 27449-2	55	1,485	44000	36703	27701	0.998
DSM 27449-3	55	1,478	29100	23668	17775	0.998
DSM 27449-4	54	1,493	28500	21813	16063	0.996
DSM 27449-5	54	1,497	36500	27780	18333	0.998
DSM 27449-6	55	1,489	31600	25899	20555	0.998
DSM 27449-7	55	1,477	34800	29490	23715	0.997
DSM 27449-8	55	1,488	41500	34528	26616	0.999

Data sources are provided by Genomics BioSci & Tech Co., New Taipei City, Taiwan. The sequencing depth was sufficient, as indicated by the Good's coverage value exceeding 0.99.

Table S2. MicroRNA stem-loop reverse transcribed primer and sequence of primers and probes for miR-Q probe qPCR Assay

Assay Name	5'-labeled	Sequence	3'-labeled
rno-miR-30a-5p reverse transcribed primer		GTCGTATCCAGTGCAGGGTCCGAGGTATTCGCACTG GATACGACCTTCCA	
rno-miR-30a-5p-F	----	CGCGTGTAAACATCCTCGAC	----
rno-miR-30a-5p-R	----	AGTGCAGGGTCCGAGGTATT	----
rno-miR-30a-5p-P	FAM	ACGACCTTCCAGTCG	MGB
rno-miR-324-3p reverse transcribed primer		GTCGTATCCAGTGCAGGGTCCGAGGTATTCGCACTG GATACGACCCAGCA	
rno-miR-324-3p-F	----	ATATCCACTGCCCCAGGTGC	----
rno-miR-324-3p-R	----	AGTGCAGGGTCCGAGGTATT	----
rno-miR-324-3p-P	FAM	ACGACCCAGCAGCA	MGB
rno-miR-93-5p reverse transcribed primer		GTCGTATCCAGTGCAGGGTCCGAGGTATTCGCACTG GATACGACCTACCT	
rno-miR-93-5p-F	----	CGCAAAGTGCTGTTCGTGC	----
rno-miR-93-5p-R	----	AGTGCAGGGTCCGAGGTATT	----
rno-miR-93-5p-P	FAM	ATACGACCTACCTGC	MGB
rno-miR-223-3p reverse transcribed primer		GTCGTATCCAGTGCAGGGTCCGAGGTATTCGCACTG GATACGACGGGGTA	
rno-miR-223-3p-F	----	GCGCGTGTCAGTTTGTCAAA	----
rno-miR-223-3p-R	----	AGTGCAGGGTCCGAGGTATT	----
rno-miR-223-3p-P	FAM	ATACGACGGGGTATT	MGB
rno-miR-320-3p reverse transcribed primer		GTCGTATCCAGTGCAGGGTCCGAGGTATTCGCACTG GATACGACTCGCCC	
rno-miR-320-3p -F	----	GCGAAAAGCTGGGTTGAGA	----
rno-miR-320-3p-R	----	AGTGCAGGGTCCGAGGTATT	----
rno-miR-320-3p-P	FAM	ACGACTCGCCCTC	MGB
rno-miR-146a-5p reverse transcribed primer		GTCGTATCCAGTGCAGGGTCCGAGGTATTCGCACTG GATACGACAACCCA	
rno-miR-146a-5p-F	----	CGCGTGAGAACTGAATTCCA	----
rno-miR-146a-5p-R	----	AGTGCAGGGTCCGAGGTATT	----
rno-miR-146a-5p-P	FAM	ACGACAACCCATG	MGB
U6 snRNA reverse transcribed primer		ATGGAACGCTTCACGAATTTG	
U6 snRNA-F	----	CTCGCTTCGGCAGCACATAT	----

U6 snRNA-R	----	ATGGAACGCTTCACGAATTTG	----
U6 snRNA-P	FAM	ACGATACAGAGAAGAT	MGB
cel-miR-39-3p reverse transcribed primer		GTCGTATCCAGTGCAGGGTCCGAGGTATTCGCACTG GATACGACCAAGCT	
cel-miR-39-3p-F	----	CGCGTCACCGGGTGTAAA	----
cel-miR-39-3p-3p-R	----	AGTGCAGGGTCCGAGGTATT	----
cel-miR-39-3p-P	FAM	ACGACCAAGCTGATTTACAC	MGB

FAM, Fluorescein amidites; MGB, minor groove binder. Data sources are provided by Topgen Biotech, Kaohsiung City, Taiwan.
